# Pentraxin 3 and biopsy status in celiac patients 

**Published:** 2018

**Authors:** Roberto Assandri, Alessandro Montanelli

**Affiliations:** 1 *Departement of Clinical Pathology, Clinical Chemistry Laboratory ASST Ospedale Maggiore di Crema, Italy*; 2 *Clinical Chemistry Laboratory, Spedali Civili di Brescia, Italy*

**Keywords:** Gluten-sensitivity, Innate immune response, Adaptive immune response, Pentraxin 3, Antigliadin antibodies, Celiac disease

## Abstract

**Aim:**

In our study we explored a possible relationship between PTX3 and CD.

**Background:**

Gluten sensitivity is known as a hallmark of celiac disease (CD). The diagnosis of CD requires demonstration of a typical enteropathy, and positive serology supports the diagnosis. The CD immune response involves the adaptive, as well as the innate immunity and is characterized by the presence of anti-gliadin (AGA) and anti-transglutaminase 2 antibodies (tTGA), lymphocytic infiltration in the intestinal epithelial membrane and expression of multiple cytokines. The long pentraxin 3 (PTX3), an acute-phase inflammatory molecule, plays an important role in innate immunity.

**Methods:**

108 CD patients were divided according to Marsh Histological grade following Marsh criteria classification in three groups: Group 1: Marsh 0, patients with a known history of CD under gluten free diet, complete remission; Group 2: Marsh1 and Marsh 2; Group 3: Marsh 3.

Healthy age-matched controls without a known history of CD or gastrointestinal symptoms (n=30) served as controls. PTX3 serum levels were measured by sandwich ELISA on an automated platform.

**Results:**

PTX3 serum levels were significantly elevated in group 3 and group 2 compared with HC (mean 3.31± 1.27 ng/mL and 3.97 ± 0.54 ng/mL versus 1.06 ± 0.59 ng/mL; *P *< 0.005), with group 1 (0.76±0.31 ng/mL). No statistically significant differences were found between group 1 and HC group. We found a strong linear correlation between PTX3 serum levels and AGA levels in group 2 (r=0.78, *P *<0.0001), and group 3 (r =0.63, *P *< 0.005) but no correlations were detected between PTX3 serum levels and tTGA levels (group 2, r= 0.04; group 3, r=0.24). Serological data revealed that PTX3 correlated with major gastrointestinal damage patients.

**Conclusion:**

PTX3 is a component of the humoral arm of the innate immune system. Our data showed that PTX3 serum levels were high in active disease patients with pathological levels of AGA. We also demonstrated that patients with normal AGA IgA levels had PTX3 serum levels compared to healthy control. We hypothesized that PTX3 is able to modulate the innate response to gliadin in CD and it could regulate the adaptive immune response.

## Introduction

 Celiac disease (CD) is considered a multiple and systemic immune-mediate disorder triggered by the ingestion of wheat gluten and related proteins (1). The HLA-DQ2 and HLA-DQ8 molecules confer susceptibility for CD by presentation of specific immunogenic gluten peptides to gluten-specific T cells in the gut (2,3). The classical CD immune response included anti-gliadin peptides (AGA) and antitransglutaminase 2 antibodies (tTGA), lymphocytic infiltration in the epithelial membrane and the lamina propria, and expression of multiple cytokines pathway (4). The role of adaptive immune response to gluten has been so far broadly discussed (5), but new evidence outline the concept of a direct involvement of the innate immune system in CD pathogenesis (6). 

Innate immune system can be induced by different fragments of gliadin. For example, IL-15 cytokine is rapidly induced after gliadin exposure only in celiac patients (7,8). Finally, the expression of non-classic MHC class I molecules in response to gluten exposure activates CD8+ cytotoxic T cells, which can target and destroy epithelial cells (9). 

Pentraxins (PTXs) are a superfamily of multifunctional molecules produced by cells involved in innate immunity such as polymorphonuclear leukocytes (10). This molecule interacts with several ligands (IL-15 or TNF-α) which play a role in innate immunity (11-14). 

Several evidence underline the concept that PTX3 might function as modulator of inflammatory processes in patients with autoimmune disorders such as systemic lupus erythematosus (SLE), rheumatoid arthritis and systemic vasculitis and the PTX3 concentration increases in correlation with disease activity (15-20). 

Based on our evidence (21) and literature review, PTX and gliadin peptides could play a role in the activation of innate immunity system and in regulation of adaptive immune response. 

In this paper we investigated the relationship between PTX3 and Marsh biopsy status in CD patients, a model of gluten-sensitivity condition. In addition, we proposed a new possible pathway of PTX3 in this clinical condition. 

## Methods

**Patients**

We examined 108 new patients with CD. The diagnosis fulfilled the criteria established by the World Gastroenterology Organization (22). 

All patients included in the present study underwent the following tests: CD sierology (Anti endomysial antibodies (EMAs), anti-tissue trasnglutaminase (tTGA) and anti deamidated gliadin (DGP-IgA class), food specific IgE).

• Small intestinal biopsy was offered to all patients on a gluten-containing diet to assess mucosal status. At least four biopsy specimens were obtained, including a specimen from the duodenal bulb. Biopsy sections were prepared from duodenal formalin-fixed, paraffin-embedded material; biopsy specimens were histologically evaluated according to the Marsh classification scheme. Immunostaining to identify intraepithelial lymphocytes IEL was performed; intraepithelial lymphocytosis was defined as the presence of >25 IELs/100 epithelial cells.

• Histological grade following Marsh criteria classification (Marsh stage 0: normal mucosa, Marsh stage 1: increased number of intra-epithelial lymphocytes, usually exceeding 20 per 100 enterocytes Marsh stage 2: proliferation of the crypts of Lieberkuhn, Marsh stage 3: partial or complete villous atrophy and crypt hypertrophy). 

Healthy age- matched individuals without a known history of CD or gastrointestinal symptom (n=30) served as controls.

***Blood samples***

Venous blood samples were collected after a minimum of 4 hours of fasting. The erythrocyte sedimentation rate (ESR) was analyzed in the local laboratory. The samples for CRP and PTX3 level analysis were collected in sterile containers without additives and centrifuged after coagulation (2 hours). The serum was stored at -75°C in small specimen containers and sent to the laboratory on dry ice. 

***Serological analysis***

Quantitative detection of DGP- IgA in human serum was assessed by indirect solid-phase enzyme immunoassay ELISA (ORGENTEC Diagnostika, Germany) test. The cut-off value was set at >10 arbitrary units. Quantitative detection of tTG IgA in human serum was assessed by an ELISA (ORGENTEC Diagnostika). The cut-off value was set at >10 arbitrary units. EMA IgA was determined by indirect immunofluorescence using monkey esophagus sections as a substrate (ORGENTEC Diagnostika). Dilutions >1:10 were considered positive and then titrated. Serum IgA levels were evaluated in all subjects by nephelometry to exclude the presence of selective IgA deficiency (ie, serum IgA concentration <10 mg/dL).

***Allergological Workup***

Serum samples were analyzed for specific IgE antibody titers against wheat and gluten using a commercially available system (Immuno CAP, Phadia 250; Phadia, Uppsala, Sweden). The cut-off values were set for values > 0.35 kU/L.

***PTX3 assay***

PTX3 levels were measured by a sandwich ELISA kit (Hycult Biotech, the Netherlands), on the automated platform DSX (Techogenetics). Each sample was tested in duplicate at a dilution of 1:4 and the reported value refers to the mean of the two determinations. Both intra- and interassay coefficients of variation (CV) did not exceed 3%. The assay did not cross-react with CRP or serum amyloid A protein. Briefly, polystyrene microplates coated with a monoclonal antibody against PTX3 were incubated with serum samples for 1 hours at 37°C. For detection, biotinylated tracer polyclonal antibody specific to PTX3 was added for 1 hour and a Streptavidin-peroxidase conjugate was added for 1h. Color reaction was developed using tetramethylbenzidine and hydrogen peroxide. Optical density at 450 nm was measured with a plate reader, and absolute values were calculated from the four-parameter logistic standard curve.

**Table 1:** Features of CD patients and healthy controls

Features	CD patients	Healthy controls	p value
**Clinical features**			
Age (years)	47 ± 5^*^	47.8 ± 5	0.08
Systolic blood pressure (mmHg)	120 ± 15	118 ± 7	0.80
Diastolic blood pressure (mmHg)	72 ± 13	70 ± 12	0.84
Hypertension (%)	1.3	1.1	0.91
Diabetes (%)	0	0	NA
BMI (Kgm^2^)	20.9 ± 2.5	23.3 ± 5.0	<0.01
*Intestinal symptoms*			
Diarrhea (n,%)	34, 31,5	/	NA
Weight loss (n,%)	15, 13,5	/	NA
Indigestion (n,%)	17, 15,7	/	NA
Constipation (n,%)	1, 1,3	/	NA
Abdominal pain (n,%)	24, 22,4	/	NA
*Extraintestinal symptoms*			
Iron-deficiency anemia (n,%)	55.4, 51.3,	/	NA
Osteoporosis (n,%)	3, 2,6	/	NA
Dermatitis herpetiforme (n,%)	1, 1,3	/	NA
**Laboratory results**			
Total cholesterol (mg/dL)	198±75	193±38	0.09
LDL cholesterol (mg/dL)	96±8	98±1	0.08
HDL cholesterol (mg/dL)	37±15	53±12	<0.05
Triglycerides (mg/dL)	48±18	50±12	0.09
Serum iron (ug/mL)	38±5	100±14	<0.05
Haemoglobin (g/dL)	9.5±1	13.4±2	<0.05
CD-related marker			
AGA	52.57±49.83	0.5±0.1	<0.05
tTGA	383.72±164.21	1±0.4	<0.05
EMA (n,%)	37, 48.6%	0, 0	<0.05
Inflammatory marker			
Erythrocyte sedimentation rate (mm/h)	29,1 ± 11	33 ± 7	0.15
C-reactive protein (mg/dL)	0.9 ± 0.7	0.7 ± 0,6	0.51
Fibrinogen (mg/dL)	178±9	176±8	0.28

* mean ± standard deviation; /= not present, AGA= Anti gliadin antibodies, tTGA= Anti tissue transglutaminase antibodies, EMA= anti endomysial antibodies

**Table 2:** CD serology markers and PTX3 concentration in patients Groups

Serological Markers	Group 1 (n=36) under gluten free diet , complete remission (mean± sd UI/mL)	Group 2 (n=36) Marsh1 and Marsh 2, active disease.	Group 3 (n=36) Marsh 3, active disease.
tTGA	0,81±0.7*	54±21	503±200
AGA	3.4±1.3	35,8±3,1	106,6±30
EMA (n/n_TOT_, %)	0	35/36, 99	36/36, 100
PTX3 serum oncentration	1.1±0,63	3.31±1.27	6.2 ±0.54

* mean ± standard deviation; AGA= Anti gliadin antibodies, tTGA= Anti tissue transglutaminase antibodies, EMA= anti endomysial antibodies

**Statistical analysis**

Demographic characteristics are presented as the mean ± SD for continuous variables and as frequencies and percentages for categorical variables. PTX3 concentrations were compared between CD patients and healthy controls by using ANOVA and Bonferroni correction. The associations between the PTX3 level, laboratory parameters, biopsy and clinical characteristics were examined in the CD patients using Rank Biserial Correlation analysis. Spearman’s rank correlation coefficients were calculated to assess univariate associations between plasma PTX3 and continuous variables. All analyses in this study employed a level of significance of p<0.05 (two-sided). 

## Results

**Background data of CD patient groups and healthy subjects**

Table 1 shows the baseline characteristics of CD patients and control subjects. In this study, patients with CD and healthy controls were matched for age (47 ± 4 years vs. 47.8 ± 5 years, p = 0.89).

Adherence of GFD was evaluated after one year of gluten elimination diet, including: CD serologic panel, (tTG IgA, EMA and DGP-IgA, total cholesterol, LDL and HDL cholesterol, triglycerides, C reactive protein (CRP), erythrocyte sedimentation rate, fibrinogen), hematologic measurements (Haemoglobin and serum iron) and clinical examination (intestinal and extraintestinal symptoms). Also GFD patients were evaluated for clinical symptoms, a self-reported visual analog grade (0, no pain, to 10, worst possible pain) and a 3-point score of extraintestinal manifestations (0 absent, 1 mild, 2 moderate, 3 severe)

Histological grade following Marsh criteria classification:

- Group 1: Marsh 0, n=36 CD patients responsive to dietary therapy, patients with a known history of CD under gluten free diet (GFD) and in complete remission.
- Group 2: Marsh1 and Marsh 2, n=36 CD patients with active disease.
- Group 3: Marsh 3, n=36 CD patients with active disease.

Clinical manifestations (intestinal and extra intestinal symptoms) were typical for CD. Iron deficiency anaemia and diarrhoea were the most common presenting clinical symptoms, affecting 60.2 % and 55.3 % of the cohort. CD patients had a lower BMI (p = 0.021) than healthy control subjects and there were no significant differences with respect to smoking, history of coronary heart disease, and postmenopausal status (women only) between the two groups (data not shown).

We investigated the lipid profile of CD patients and control subjects. The mean total cholesterol at diagnosis of CD was 198±45 mg/dL and HDL- cholesterol was 37±25 mg/dL (Table 1). There was no statistical difference with respect to the classical markers of inflammation C reactive protein (CRP), Erythrocyte sedimentation rate (ESR) and fibrinogen but CD patients had a lower levels of haemoglobin and serum iron (p<0.05).All groups were characterized by the same number of patients (n=36). 

Table 2 summarized the mean tTG AGA mean value, the percentage of endomysial antibody (EMA)-positive patients and PTX3 serum levels. Statistically significant differences were present between group 1, 2 and group in 3 tTGA serum levels (0.81±0.7 UI/mL ; 54±21 UI/mL and ; 503±200 respectively p<0.05), AGA serum levels (3.4±1.3; 70 UI/mL 35.8±3.1 UI/mL; 102.6±30 UI/mL respectively) and EMA (group 1 0/36, group 35/36, 97.2 ,% ; group 3 36/36,100%). 

**Serum PTX3 concentration**

***PTX3 serum levels and*** ***small-bowel mucosal biopsies status***

We had previously categorised small bowel biopsies status of CD patients according to Marsh classification and we compared serum PTX3 levels between the groups.

Serum PTX3 concentration in sera of patients groups and control subjects group were measured by ELISA:

- In group 1 PTX3 serum levels ranged from 0,68 to 1,80 mg/dL with a mean ± SD: 1.1±0,63 ng/mL and a median value of 1,21 mg/dL.
- In group 2 PTX3 serum levels ranged from 1.12 to 5.47 mg/dL with a mean ± SD: 3.31±1.27 ng/mL and a median value of 3.08 mg/dL.
- In group 3 with active CD patients PTX3 plasma levels ranged from 2.1 to 8.04 ng/mL, with a mean ± SD of 6.2 ±0.54 ng/mL and a median value of 3.91 ng/mL.
- In the control subjects, PTX3 ranged from 0.54 to 1.67 ng/mL, with a mean ± SD of 1.06±0.59ng/mL and a median value of 1.08 ng/mL.

PTX3 levels were significantly elevated in group 2 and group 3 compared to controls, and group 1 (p<0.05). There was statistical difference in PTX3 serum level between group 1 and 2 (p<0.05). 

***PTX3 serum levels, and their correlations***

We examined correlations between serum PTX3 concentration, clinical status, CD-related markers and other laboratory parameters in the patients with CD (Table 3).

**Table 3:** Correlations between the serum PTX3 concentration, clinical status, CD-related markers and other laboratory parameters in CD patients. Significance values were calculated using Rank Biserial Correlation

Variables	Correlation coefficientt	p value
**Clinical features**		
Age (years)	0.03	0.82
BMI (Kgm^2^)	0.15	0.84
*Intestinal symptoms*		
Diarrhea (n,%)	0.17	0.31
Weight loss (n,%)	0.03	0.78
Indigestion (n,%)	0.07	0.6
Constipation (n,%)	0.17	0.31
Abdominal pain (n,%)	0.08	0.48
*Extraintestinal symptoms*		
Iron-deficiency anemia (n,%)	0.57	<0.05
Osteoporosis (n,%)	0.02	0.91
Dermatitis herpetiforme (n,%)	0.02	0.89
**Laboratory results**		
Total cholesterol (mg/dL)	0.13	0.29
LDL cholesterol (mg/dL)	0.14	0.24
HDL cholesterol (mg/dL)	0.19	0.21
Triglycerides (mg/dL)	0.289	<0.05
Serum iron (ug/mL)	- 0.65	<0.05
Haemoglobin (g/dL)	- 0.61	<0.05
Inflammatory marker		
Erythrocyte sedimentation rate (mm/h)	0.06	0.66
C-reactive protein (mg/dL)	0.07	0.43
Fibrinogen (mg/dL)	0.07	0.43

We did not identify any significant differences in serum PTX3 levels in relation to clinical symptoms with exception of anemia (p<0.001). There was no correlation between the PTX3 concentration and various coronary risk factors, including age, blood pressure and BMI.

**Figure 1:**
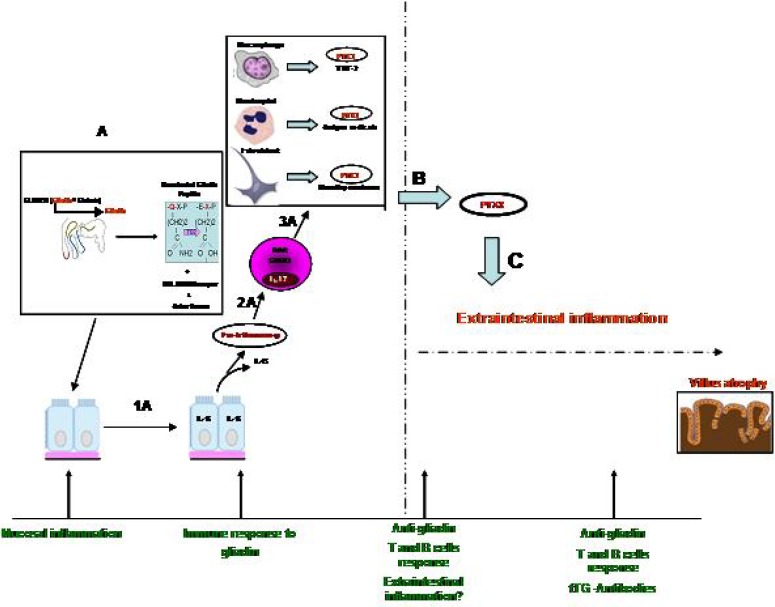
The mechanism of PTX3

We also investigated whether PTX3 serum levels were associated with CD-related serological markers. 

We found a linear correlation between PTX3 serum levels and AGA levels in group 1 (r=0.78, *p=*0.0006), group 2 (r =0.63, *p=*0.003) but no correlation was detected between PTX3 serum levels and tTGA levels in any group of patients with active disease (group 2, r= 0.04, p<0.05; group 3, r=0.24, p<0.05 and consequently tested using two tail *t* test. No statistically significant differences were found between two groups (p=0.81).

Finally, we have investigated correlations between PTX3 serum levels and certain routine laboratory parameters. No correlation were present between PTX3 and serum cholesterol levels (total cholesterol, HDL cholesterol, LDL cholesterol) but PTX3 was positively correlated with triglycerides (r = 0.289, p = 0.029) and negatively correlated with hemoglobin (r = -0.31, p=0.006) and serum iron (r= -0.25, p=0.02). 

Finally, we investigated the possible correlation between serum PTX3 and classical inflammatory markers. Serum PTX3 levels did not show a significant correlation with CRP, ESR and fibrinogen (Table 4). 

## Discussion

The pathogenesis of CD involves a complex interplay between environmental, genetic, and immunologic factors. Wheat gluten and related proteins elicit immune responses in the small intestine and drive extra intestinal manifestations (9). Genes encoding class II human leukocyte antigens HLA-DQ2 and -DQ8 are linked to the disease and are found in nearly all celiac patients (3). 

The immunologic response to gluten includes antibody reactivity to gluten proteins and autoantigens TG2, CD4+ T cell reactivity to gluten, intraepithelial CD8+ T cells, and a number of cytokines and chemokines (9), (22). Our recent work (21) has taken into consideration that PTX3 has multiple regulatory roles on innate immunity. 

It is now known that PTX3 modulates opsonisation (12), complement activation (23), and leukocyte recruitment and activation (24,25), all processes that affect autoimmune tissue injury. 

In the light on our recent data and literature review, we wanted to re-investigate the role of PTX3 in CD. 

In our paper, data showed and remarked that PTX serum level was high in patients with active disease, especially in subjects with AGA IgA pathological levels. Our previous data also demonstrated that active-CD patients with normal AGA IgA levels had a PTX3 serum level comparable to healthy control or to inactive-CD patient (21). In this work we showed that serological data revealed that PTX3 correlated with Marsh classification criteria. In addition, PTX3 serum levels directly correlate with the major extraintestinal manifestation such as anemia. Finally, GFD patients revealed a lower PTX3 serum levels than active patients. 

It is now known that antigens have to be recognized in the context of an activated innate immune system to induce sufficient adaptive immune responses (26). The adaptive immune response is initiated by APCs, primarily dendritic cells (DCs) but also macrophages and B cell subsets, which present to T-cell antigenic fragments in complex with cell surface MHC class II molecules (27) Gliadin-specific T-cell responses have been found to be enhanced by the action of intestinal transglutaminases. The infiltration of T cells in the lamina propria of the active celiac lesion is dominated by CD4+ memory T cells (CD45RO+) bearing the α/β T-cell receptor (TCR) (27). In our recent editorial we hypothesized that PTX3 could be overexpressed during gliadin and gluten exposure and modulate intestinal immune response to gluten and relate proteins by the pathway that involve NF-kB and TLR4 (28).

Based on literature review and our data (21), we can elaborate a hypothetical mechanism of PTX3 mediated molecule in gluten sensitivity pathogenesis: ingestions of gluten in susceptible individual can trigger the immune response to gliadin peptides and may induce activation of CD4+ T cells, which in relation to the cytokine microenvironment may develop into CD4+ Th17 subpopulation cells. IL-15 could activate the innate immune response to gliadin peptides and could stimulate the production of PTX3 in three cell types involved in this mechanism (macrophage, neutrophil and fibroblast,). It is now known that PTX3 is produced by these cells in several pathological environments (23) and, together with CD4+ Th1 cells, have a potent of pro-inflammatory effect. Based on literature review, PTX3 can also modulate the adaptive immune response, could support the maintenance of adaptive cells and drive the extraintestinal manifestation, as demonstrated by our data. Furthermore, a recent Chinese work showed that Lactobacillus acidophilus could transiently regulate the immunity and inflammatory mediator factor PTX3 expression through rapidly activating NF-kappa-B signalling pathway in intestinal epithelial cells. In CD scenario, PTX3-RNA could be ever overexpressed and modulate intestinal immune response to gluten and relate proteins (29). Our study showed that PTX3 serum concentration increased in patients with major bowel damage (Marsh score). Figure 1 showed a hypothetical mechanism. CD with villous atrophy is at the end of a spectrum, being characterized by gluten-induced innate immunity. 

Correlation between AGA and PTX3 serum levels acquired some interest if we considered that many patients with non-celiac gluten sensitivity (NCGS) displayed an elevated prevalence of high titre, “first-generation” IgG AGA directed against native gliadin (56.4%) (24). NCGS, originally described in the 1980s, is a “re-discovered” disorder characterized by intestinal and extra-intestinal symptoms related to the ingestion of gluten-containing food, in subjects that are not affected with either CD or wheat allergy (WA) (25). So far no specific biomarker of NCGS has been identified.

The prevalence of IgG AGA detected in NCGS, although lower than that found in CD (81.2%), was much higher than other pathologic conditions such as connective tissue disorders (9%) and autoimmune liver diseases (21.5%) as well as in the general population and healthy blood donors (2%–8%). On the other hand, the prevalence of IgA AGA in NCGS patients was very low (7.7%).

The “best” CD markers, namely IgG deamidated gliadin peptide (DGP) antibodies, IgA tTGA, and IgA EMA, were always negative in NCGS patients, except for an isolated positivity at a very low titre for IgG DGP (30-32). 

We suggested an interesting hypothesis in which PTX3 could ever be overexpressed during gliadin and gluten exposure and modulate intestinal immune response to gluten and relate proteins by the pathway that involve NF-kB and TLR4. Both innate and adaptive immunity have a central role in the development of CD, gluten sensitivity seems to be mainly associated with activation of the innate immune response (26). In this interesting scenario our study supports the hypothesis that PTX3 could perform a critical role as mediator of inflammation in several steps that link toxic gliadin ingestion and tissue damage.

## Conflict of interests

The authors declare that they have no conflict of interest.
